# Regulation of Axonal HCN1 Trafficking in Perforant Path Involves Expression of Specific TRIP8b Isoforms

**DOI:** 10.1371/journal.pone.0032181

**Published:** 2012-02-21

**Authors:** Wiebke Wilkars, Zhiqiang Liu, Alan S. Lewis, Travis R. Stoub, Elena M. Ramos, Nicola Brandt, Daniel A. Nicholson, Dane M. Chetkovich, Roland A. Bender

**Affiliations:** 1 Institute of Neuroanatomy, University of Hamburg, Medical Center, Hamburg, Germany; 2 Davee Department of Neurology and Clinical Neurosciences, Northwestern University Feinberg School of Medicine, Chicago, Illinois, United States of America; 3 Department of Neurological Sciences, Rush University Medical Center, Chicago, Illinois, United States of America; 4 Department of Physiology, Northwestern University Feinberg School of Medicine, Chicago, Illinois, United States of America; University of Muenster, Medical Center, Germany

## Abstract

The functions of HCN channels in neurons depend critically on their subcellular localization, requiring fine-tuned machinery that regulates subcellular channel trafficking. Here we provide evidence that regulatory mechanisms governing axonal HCN channel trafficking involve association of the channels with specific isoforms of the auxiliary subunit TRIP8b. In the medial perforant path, which normally contains HCN1 channels in axon terminals in immature but not in adult rodents, we found axonal HCN1 significantly increased in adult mice lacking TRIP8b (TRIP8b^−/−^). Interestingly, adult mice harboring a mutation that results in expression of only the two most abundant TRIP8b isoforms (TRIP8b[1b/2]^−/−^) exhibited an HCN1 expression pattern similar to wildtype mice, suggesting that presence of one or both of these isoforms (TRIP8b(1a), TRIP8b(1a-4)) prevents HCN1 from being transported to medial perforant path axons in adult mice. Concordantly, expression analyses demonstrated a strong increase of expression of both TRIP8b isoforms in rat entorhinal cortex with age. However, when overexpressed in cultured entorhinal neurons of rats, TRIP8b(1a), but not TRIP8b(1a-4), altered substantially the subcellular distribution of HCN1 by promoting somatodendritic and reducing axonal expression of the channels. Taken together, we conclude that TRIP8b isoforms are important regulators of HCN1 trafficking in entorhinal neurons and that the alternatively-spliced isoform TRIP8b(1a) could be responsible for the age-dependent redistribution of HCN channels out of perforant path axon terminals.

## Introduction

Hyperpolarization-activated cyclic nucleotide-gated (HCN) channels, which generate the h-current (I_h_), are involved in a variety of important neuronal functions such as contributing to the resting membrane potential, generating rhythmic activity and regulating temporal summation of synaptic input [1], [2], [3]. Physiological properties of HCN channels are largely determined by their subunit composition, which includes four pore-forming subunits (HCN1-4) [4], [5], [6], [7], and in neurons, an auxiliary subunit, tetratricopeptide repeat (TPR)-containing Rab8b-interacting protein (TRIP8b) [8], [9], [10], [11], [12]. The function of HCN channels in neurons further depends critically on their subcellular localization, which can vary significantly among neuronal types and may include somatic, dendritic as well as axonal compartments [2], [3], [13]. The factors that regulate the transport of HCN channels to subcellular compartments in a neuron-type-specific manner are largely unknown, but recent findings from genetically-modified mice [12], [14] suggest that TRIP8b is critical for proper HCN channel trafficking. TRIP8b interacts with HCN1-4 subunits at two distinct sites to control channel gating and surface membrane expression [9], [15], [16], and in the absence of TRIP8b, the normal localization of HCN channels in distal dendrites of cortical and hippocampal area CA1 pyramidal neurons is disrupted [12]. Because alternative splicing of TRIP8b leads to expression of distinct isoforms with different effects on HCN channel trafficking to the surface plasma membrane [8], [9], [10], we reasoned that distinct TRIP8b isoforms might contribute to distinct HCN channel expression patterns in different neurons.

We previously showed that in the medial perforant path (mPP), a major pathway connecting entorhinal cortex (EC) and hippocampus, HCN1 is expressed in axon terminals in an age-dependent manner. Specifically, HCN1 is expressed in mPP axons in immature rodents, but this expression decreases with maturation, resulting in age-dependent changes in the properties of synaptic transmission [17]. This age-dependent decrease of axonal HCN1 in mPP is not associated with a down-regulation of HCN1 mRNA or protein expression in the cells of origin - the layer II stellate cells of the medial EC -, suggesting that its regulation involves post-translational mechanisms such as association with auxiliary proteins [17]. Using TRIP8b-deficient mice, EC tissue and entorhinal neuron culture for analysis of TRIP8b effects on HCN1 expression, we here provide evidence that TRIP8b is involved in the developmental regulation of axonal HCN channel expression, and that a specific TRIP8b isoform - TRIP8b(1a) - may be particularly important for the regulation of axonal HCN1 transport in perforant path with age.

## Materials and Methods

### Ethics statement

All animal experiments were performed according to legal guidelines (U.S. resp. German law) and were approved by the institutional committees for the care and use of laboratory animals (Northwestern University Institutional Animal Care and Use Committee: protocol No. 2010-0571; University Hamburg Animal Care Committee: protocol Nos. ORG_471 and ORG_472). Animals were maintained on a 12 h light/dark cycle and were provided with food and water ad libitum.

### Generation of knockout mice

The generation of mice with a total body elimination of TRIP8b (TRIP8b^−/−^) has been described in detail previously [12]. Partially TRIP8b-deficient mice (Pex5l[1b/2]^−/−^) were generated from ES cells prepared by Regeneron for the NIH Knockout Mouse Project. Details of the ES cells can be found at www.komp.org. Briefly, a targeting vector (project ID VG11153) was used to generate the allele Pex5l^tm1(KOMP)Vlcg^, in which exons 1b and 2 of the gene encoding TRIP8b (*Pex5l*) are replaced by the lacZ coding sequence (inserted directly after the start codon in exon 1b), followed by a neomycin selection cassette flanked by *lox*P sites (Supplementary Fig. s1). Homologous recombination generated neomycin-resistant VGB6 KOMP ES cell lines (Regeneron) carrying the Pex5l^tm1(KOMP)Vlcg^ allele on the C57BL/6 genetic background (for clarity and for consistency in nomenclature, this allele is referred to hereafter as TRIP8b[1b/2]^−/−^). Blastocyst injections of these ES cell lines into pseudopregnant B6(Cg)-*Tyr^c-2J^*/J mice were performed at the Northwestern University Transgenesis and Targeted Mutagenesis Lab and produced chimeric offspring that were bred with C57BL/6 albino females B6(Cg)-*Tyr^c-2J^*/J. Black offspring were genotyped to detect the targeted allele. Heterozygous offspring TRIP8b[1b/2]+/− were crossed with heterozygous siblings to yield homozygous mice (TRIP8b[1b/2]−/−). For genotyping the TRIP8b[1b/2]−/−) and wildtype alleles, tail genome DNA was extracted, and the following primers were employed, 5′-TCATTCTCAGTATTGTTTTGCC-3′ (NeoFwd, for [1b/2]−/−), 5′-AGA GAA CTG CTT GTT TAT CTC-3′ (WtFwd, for wild type) and 5′-TGACTCATTGCATTGAGTCC-3′ (SD for both). PCR reaction conditions are as follows: Initial denaturing at 95°C for 3 minutes, denaturing at 95°C for 30 seconds, annealing at 50°C for 45 seconds, extension at 72°C for 2 minutes. Steps from denaturing to extension were run for 36 cycles and included a final extension at 72°C for 5 minutes. The PCR products were separated with 2% agarose gels. TRIP8b[1b/2]^+/+^ is indicated by a 626 bp band, TRIP8b[1b/2]^−/−^ is 400 bp and TRIP8b[1b/2]^+/−^ contains both.

### Immunofluorescence and immunohistochemistry

For immunohistochemistry, adult (>postnatal day [P] 50) mice (n = 4) or rats (n = 3) were deeply anesthetized with ketamine/xylazine (12 mg/ml ketamine and 1.6 mg/ml xylazine in saline, i. p.), then transcardially perfused with phosphate-buffered saline (PBS) followed by ice-cold 4% paraformaldehyde (PFA). Brains were removed, postfixed in 4% PFA (overnight) and transferred to 30% sucrose solution (24–48 h). Brains were then sectioned into 30 µm slices on a Leica microtome. For HCN1 and TRIP8b double-fluorescent labeling of mouse slices, antigen retrieval was performed by treatment of slices with 10 mM sodium citrate buffer, pH 9.0, for 10 min at 80°C. Slices were blocked in 5% normal goat serum and 5% normal donkey serum in PBS plus 0.03% Triton X-100 for 30 min and incubated overnight in primary antibodies (guinea pig polyclonal anti-HCN1, 1∶1,000 [18]; mouse monoclonal anti-TRIP8b, 1∶10 [19]; both diluted in blocking solution). Slices were rinsed and incubated in 1∶1,000 fluorescence-conjugated donkey anti-mouse IgG and goat anti-guinea pig IgG secondary antibodies (diluted in blocking buffer) for 2 h. Slices were then mounted on glass slides, using PermaFluor mounting reagent (Thermo Fisher Scientific) and coverslipped. For light microscopy (TRIP8b expression in rat), sections were incubated with rabbit anti-TRIP8b antibody (1∶10,000 [20]) overnight, labeled with biotin-conjugated goat anti-rabbit IgG secondary antibody and then subjected to biotin-avidin-peroxidase-complex solution (ABC-Kit, Vector Laboratories). TRIP8b immunoreactivity was visualized by incubating sections in a solution containing 0.04% 3, 3′ diaminobenzidine, 0.01% H_2_O_2_ and 0.01% NiCl_2_. Finally, sections were dehydrated in a graded ethanol series and coverslipped with Entellan (Merck).

### Imaging and quantitative immunofluorescence analysis in mouse dentate gyrus

**Figure 1:**
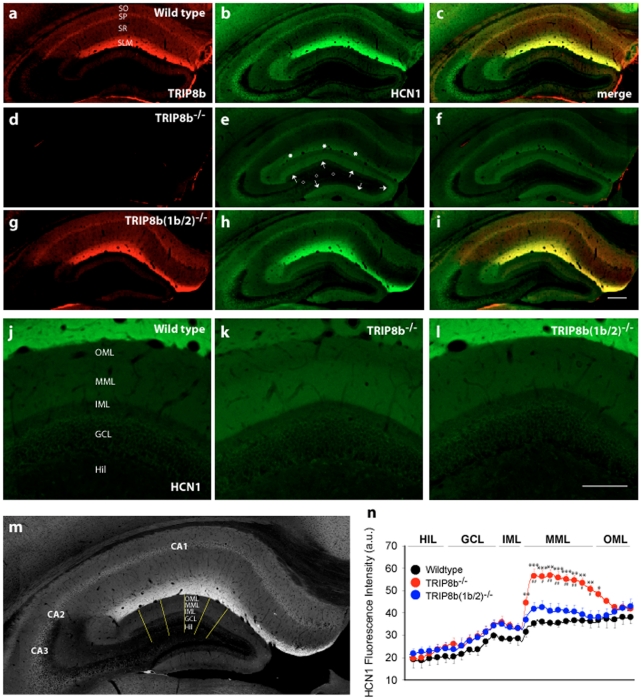
Differential effects of TRIP8b isoforms on localization of HCN1 in the middle molecular layer (MML) of dentate gyrus. a–c) Representative low power images of coronal sections of adult wildtype mouse brain show the characteristic distribution of TRIP8b (a) and HCN1 (b) immunoreactivity in hippocampus, with HCN1 and TRIP8b staining most intense in stratum lacunosum-moleculare (SLM) of CA1, and low expression in stratum pyramidale (SP), stratum oriens (SO) and stratum radiatum (SR). In contrast, much lower levels of HCN1 and TRIP8b are expressed in hippocampal dentate gyrus (DG). d–f) Interestingly, in adult TRIP8b^−/−^ mice the immunoreactivity of HCN1 is increased in the MML of DG (arrows in e), where the granule cells are innervated by axons from medial EC via the perforant path. No changes in HCN1 expression were observed in mossy fibres (empty circles), whereas there is significant reduction of HCN1 staining in the SLM of CA1 (asterisks). g–i) Adult TRIP8b[1b/2]^−/−^ mice expressing TRIP8b isoforms 1a and 1a-4 show similar HCN1 staining patterns to wildtype mice in all hippocampal subregions. Scale bar: 200 µm. j–l) Higher magnification views of HCN1 immunoreactivity in TRIP8b^−/−^ DG clearly show the increased HCN1 staining in MML (k) as compared with wildtype (j) and TRIP8b[1b/2]^−/−^ mice (l). Scale bar: 50 µm. m–n) HCN1 immunofluorescence was measured as a function of distance across the superior blade of the dentate gyrus as indicated in the diagram (m), and demonstrates higher HCN1 expression in MML in TRIP8b^−/−^ (red circles) as compared to wildtype (black circles) and TRIP8b[1b/2]^−/−^ (blue circles) mice (n). OML - outer molecular layer, MML - middle molecular layer, IML - inner molecular layer, GCL - granule cell layer, Hil - hilus. Asterisks in (n) denote statistical significance (*p<0.05, **p<0.01, ***p<0.001).

Low power images were obtained on a TissueGnostics (Vienna, Austria) imaging system. Briefly, to define imaging area, the slide was previewed with an EC Plan-Neofluar 2.5× objective (NA 0.075) under single labeling for HCN1. Then imaging parameters for wildtype HCN1 and TRIP8b fluorescence labeling signals were entered for EC Plan-Neofluar 20× objective (NA 0.5; fixed X and Y speed, acceleration, automatic focus in the 300 µm range around the focus as it was in Z axis, scanning mode, exposure time and digital offset). Next, images used for quantitative analysis were acquired with the 20× objective by the same parameter settings. The quantification of HCN1 immunoreactivity was performed as previously described with some modification [12], [18]. For quantification of HCN1 immunoreactivity in hippocampal DG, we first selected an appropriate area in DG with clear borders between adjacent subregions (hilus - HIL, granule cell layer - GCL, inner molecular layer - IML, middle molecular layer - MML and outer molecular layer - OML; see Fig. 1m). Using NIH Image J software [21], the fluorescence intensity along 5 perpendicular lines (150 µm interval) spanning HIL, GCL, IML, MML and OML were measured. The points across the border between GCL and IML were established as the references for centering the data from the different lines. The pixel fluorescence intensity at each distance from the reference was then averaged. For statistical analysis, fluorescence intensity was averaged for 10 pixel segments along the 5 lines crossing the dentate gyrus and plotted as intensity versus distance from granule cell layer. Data are expressed as mean ± SEM. The statistical comparisons of wildtype, TRIP8b^−/−^ and TRIP8b(1b/2)^−/−^ were performed using one-way ANOVA followed by Tukey's *post hoc* test. Data were considered statistically significant if *p*<0.05.

### Preembedding ultrasmall silver-intensified immunogold electron microscopy

All electron microscopy reagents unless otherwise specified were from Electron Microscopy Sciences. Four mice (two wildtype and two TRIP8b^−/−^ adult littermates) were deeply anesthetized by isoflurane (Isothesia, Butler) and transcardially perfused with 0.9% saline followed by ∼50 ml ice-cold acidic fixative (2% paraformaldehyde, 1% glutaraldehyde in 0.1 M sodium acetate buffer, pH 6.0) followed by a slow perfusion of ice-cold basic fixative (2% paraformaldehyde, 1% glutaraldehyde in 0.1 M sodium borate buffer, pH 9.0) for 1 h. At the conclusion of the perfusion, brains were removed and placed in ice-cold basic fixative overnight at 4°C on a shaker. The next day brains were cut into two hemispheres, rinsed 3×20 min in 0.12 M TBS and cut into 70 µm coronal sections on a vibratome. Sections were rinsed in TBS 5×5 min and treated with 1% NaBH_4_ in TBS for 30 min. Sections were rinsed in TBS 5×1 min and incubated in blocking solution (10% NGS in TBS) for 30 min followed by incubation in primary antibody overnight at 4°C. Primary antibody (guinea pig anti-HCN1, 1∶1,000 [18]) was diluted in 2% NGS+0.1% or 0.5% Triton X-100 in TBS. The next day slices were rinsed in incubation buffer 1×5 min and in TBS 10×5 min. Slices were incubated in secondary blocking buffer (2% NGS+1%BSA+0.3% cold water fish skin gelatin in TBS) for 1 h followed by incubation in ultrasmall immunogold (Aurion) anti-guinea pig secondary antibody 1∶100 dilution in 2% NGS+1% BSA-C+0.3% CWFSG at 4°C for ∼40 h. Slices were then rinsed in incubation buffer 1×5 min, TBS 6×10 min, PBS 2×5 min followed by fixation of immunogold with 2% glutaraldehyde in PBS for 1 hr. Slices were rinsed 2×5 min in PBS, 4×10 min in TBS, followed by enhancement conditioning solution (ECS) 3×10 min. Slices were then incubated in R-Gent SE-EM Plus enhancement mixture for 90 min and rinsed in ECS 4×10 min, TBS 2×10 min, and PBS 2×10 min. Slices were osmicated with 0.4% OsO_4_ in PBS for 15 min and rinsed in PBS 3×10 min and dH_2_O 2×5 min. Slices were stained in 1% aqueous uranyl acetate for 10 min and rinsed 3×10 min in dH_2_O followed by dehydration in graded ethanols and propylene oxide. Tissue was infiltrated by a 1∶1 mixture of araldite∶propylene oxide overnight at room temperature followed by flat embedding between aclar sheets and curing for 48 h at 60°C. Tissue regions of interest were subdissected and reembedded in Araldite and cured overnight at 60°C. 65 nm ultrathin sections were cut with a diamond knife and placed onto a formvar-coated slotted grid Ultrathin sections were then stained with uranyl acetate-lead citrate (for 15 and 10 min, respectively), rinsed in ultrapure dH_2_O, and allowed to dry at room temperature.

For image acquisition and quantitative analysis, each serial ultrathin section spanned from the granule cell layer to the hippocampal fissure, encompassing the entire molecular layer of the upper and lower blades. Images were obtained with a JEOL 1200EX electron microscope. The middle molecular layer was identified using the field delineator of the electron microscope. The distance between the granule cell layer and the hippocampal fissure was determined, and the middle molecular layer was estimated to lay half-way between. Electron micrographs were obtained from 12–30 serial sections, and axospinous synapses were identified between 3 and 8 microns from the tissue surface (i.e., the surface of the 70-µm-section that was exposed to the reagents during labeling). For quantification, twenty-five asymmetric axospinous synapses were identified from each mouse, and the presynaptic bouton was followed through the serial sections. Presynaptic boutons showed a range of immunoreactivity to HCN1, ranging from immunonegative (zero particles) to highly immunopositive (e.g., in TRIP8b^−/−^ mice, some boutons had more than 12 particles). Particles bound within the presynaptic bouton were counted for each bouton, and its immunoreactivity was estimated as particles per bouton (though most in the wildtype mice were immunonegative).

### Neuron culture and transfection

With slight modifications, primary entorhinal cortex neurons were prepared from Wistar rat pups according to [22]. Cells were cultured on poly-L-lysine-coated glass coverslips (0.1 mg/ml) in neurobasal A medium, supplemented with 1% B27, 0,5 mM glutamine and the antibiotics penicillin and streptomycin (1×; all components from Invitrogen) at a density of 120.000 cells/well [23]. No glia co-cultures were used. Two different culturing approaches were applied: 1) “Immature cultures” were prepared from newborn rats (postnatal day [P] 0), single- or co-transfected with HCN1-EGFP, TRIP8b(1a) or TRIP8b(1a-4) after two days *in vitro*, and fixed in 4% PFA two days later (remaining a total of 4 days *in vitro*). 2) “Differentiated cultures” were prepared from five-day-old (P5) rats, single-transfected with TRIP8b(1a) or TRIP8b(1a-4) after seven days *in vitro*, and fixed three days later (remaining a total of 10 days *in vitro*). Transfections were carried out using Effectene (Qiagen) according to the manufacturer's instructions.

### Constructs

The generation of constructs containing C-terminally EGFP-tagged HCN1, TRIP8b(1a) or TRIP8b(1a-4) cDNA is described in detail in [9]. Mouse HCN1 cDNA was kindly provided by Drs. Bina Santoro and Steven Siegelbaum (Columbia University, New York, NY).

### Immunofluorescence in entorhinal neuron culture

The following antibodies were used for single-, double- or triple-immunostaining of rat entorhinal neuron culture: mouse monoclonal anti-glial fibrillary acidic protein (GFAP, 1∶500; Sigma), rabbit polyclonal anti-HCN1 (1∶600; Millipore), mouse monoclonal anti-MAP2 (1∶1,000; Millipore), mouse monoclonal anti-reelin (1∶500; Millipore), mouse monoclonal anti-Tau-1 (1∶1,000; Millipore), chicken polyclonal anti-Tau (1∶1,500; Abcam), rabbit polyclonal anti-TRIP8b (1∶10,000 [20]), mouse monoclonal anti-TRIP8b, constant region (1∶1,000; NeuroMab). Generally, cells were first blocked in PBS supplemented with 5% fetal calf serum after fixation, then permeabilized in PBS containing 0.2% Triton X-100. Incubation with the primary antibody was performed for 2 h at room temperature in blocking solution. After being washed with PBS containing 0.1% Triton X-100, the cells were incubated with secondary antibodies (Alexa488-conjugated goat anti-rabbit IgG, 1∶500, Cy3-conjugated goat anti-rabbit IgG, 1∶800, or Cy3-conjugated goat anti-mouse IgG, 1∶800; Molecular Probes) in blocking solution for 1 h at room temperature. If triple-labeling was intended (for Tau-staining), cells were washed again thoroughly with PBS/0.1% Triton X-100, then incubated with either Cy5-conjugated rabbit anti-mouse IgG or rabbit anti chicken IgY antibodies (1∶500; Dianova). Finally, cells were counterstained with 4′, 6-diamidin-2-phenylindol (DAPI), then mounted on glass slides and embedded with fluorescence-protecting mounting medium (Dako). Different antibody combinations were used for the triple-labeling of “immature” and “differentiated” cultures: for “immature cultures”, expressing HCN1-EGFP, rabbit polyclonal anti-TRIP8b and mouse monoclonal anti-Tau-1 antibodies were co-applied (and visualized by corresponding Cy3- and Cy5-conjugated secondary antibodies, respectively). For “differentiated cultures”, rabbit polyclonal anti-HCN1 was used to detect endogenous HCN1, and mouse monoclonal anti-TRIP8b and chicken polyclonal anti-Tau antibodies were co-applied (and detected with Alexa488-, Cy3- and Cy5-conjugated secondary antibodies, respectively).

### Imaging and quantitative immunofluorescence analysis of entorhinal neuron cultures

For quantitative analysis, double- or triple-labeled cells were screened for expression of endogenous HCN1 (differentiated cultures), HCN1-EGFP (in “immature cultures”) and TRIP8b. Cells with clear neuronal morphology (i. e. dendrites or an axon identifiable) were photographically captured using a Zeiss LSM 510 Meta confocal microscope. From those, neurons with a clearly recognizable axonal extension were further selected for an analysis of signal intensity and distribution (n = 25 for each experimental group). For this analysis, the neuron was centered in a 400 µm^2^ square with four subdivisions of 100 µm^2^. The axon hillock was always placed in the lower right subdivision, which was defined as “subdivision 1”. The others were numbered clockwise as subdivisions 2, 3 or 4, respectively. In each subdivision, the cytoplasmic area was marked (i. e., the nucleus was carefully avoided) and HCN1 or TRIP8b signal intensity (average value) was determined within the marked area using NIH Image J software [21]. In order to normalize the data, the mean value was calculated from the four subdivisional intensity values and each value was then presented as “percent of the mean”, thus reflecting relative enrichment (if larger than 100%) or depletion (if less than 100%) of the signal in the respective subdivision. Finally, for each subdivision, the data from the different experimental groups (TRIP8b(1a) vs. TRIP8b(1a-4) transfection) were compared and statistically analyzed using unpaired Student's t-tests. Error bars indicate “standard error of the mean (SEM)”. Significance level was set to p<0.05.

### In situ hybridization

For *in situ* hybridization, adult rats (>P50, n = 3) were used. Perfused brains (see above) were cut horizontally on a cryotome (30 µm), and sections collected in 2× SSC (0.3 M sodium chloride+0.03 M sodium citrate). *In situ* hybridization was performed at 47°C (hybridization) and 57°C (washes) following the protocol described previously [24]. Antisense and sense riboprobes were generated from a pBS(SK) transcription vector containing 456 bp of rat TRIP8b cDNA (encoding amino acids 60–211 of isoform 1a). They were labeled with digoxigenin (3.5∶6.5 digoxigenin-UTP/UTP) according to the manufacturer's instructions (Roche Diagnostics). To characterize TRIP8b-expressing cells in EC in more detail, some sections were further processed for immunohistochemistry after the *in situ* hybridization had been completed. These sections were incubated with mouse monoclonal anti-reelin antibody (1∶500, Millipore) for 48 h at 4°C and were then subjected after several washes to Alexa488-conjugated goat anti-mouse IgG secondary antibody for 3 h at room temparature. After DAPI-counterstaining and final washes, the sections were mounted on glass slides and embedded with fluorescence-protecting mounting medium (Dako). Photographs were taken with a Zeiss Axioskop 2 fluorescence microscope.

### Western Blots

For Western Blot analyses, entorhinal cortex tissue from six immature (P10) and six adult rats was used. Entorhinal cortices were explanted as described previously [17]. Tissue was manually homogenized (15% w/t) immediately after dissection, with a buffer containing 50 mM Tris-HCl (pH 7.4), 150 mM NaCl, 1% Nonidet-40, 0.1% sodium dodecyl sulfate, 0.5% sodium desoxycholate, 5 mM EDTA, and a mixture of protease inhibitors (Roche). The homogenates were centrifuged with 20,000× g at 4°C for 10 min, and the supernatant was collected and frozen until further use. Protein concentrations were determined with the Bradford protein assay (Bio-Rad). For analysis, extracts were run on 10% SDS-PAGE under denaturing conditions. Samples were boiled for 5 min, briefly cooled on ice, and then seperated at voltage that prevented excessive heat. Proteins were blotted on nitrocellulose membranes, blots were treated with 5% milk powder solution (in PBS+0.3% Triton-X100), and incubated with polyclonal rabbit anti-HCN1 (1∶200, Millipore) or polyclonal rabbit anti-TRIP8b (1∶10,000 [20]) antibodies overnight at 4°C. Antibody binding was detected using ECL Western blotting substrate or SuperSignal West Pico Chemiluminescent Substrate (Thermo), and quantified by densitometry using “Image J” [21]. Actin expression was determined as an internal standard using polyclonal rabbit anti-actin (1∶10,000; Sigma). Data were statistically analyzed using unpaired Student's t-test and presented as “percent of immature expression” (significance level: *p*<0.05). Further, for control purposes, recombinant TRIP8b (1a) and (1a-4) was expressed and isolated from HEK293 cells according the protocol of Lewis et al. [9].

## Results

### Enhanced expression of HCN1 in DG molecular layer of TRIP8b-deficient (TRIP8b^−/−^) mice

We recently demonstrated that the characteristic distal dendritic distribution of HCN1 in cortical and hippocampal CA1 pyramidal neurons requires the presence of TRIP8b [12] (see also Fig. 1). Despite the fact that HCN1 protein levels are reduced by 40% in area CA1 [12] we find that HCN1 expression in the dentate gyrus is increased in adult TRIP8b^−/−^ compared to wildtype mice (Fig. 1a–f; arrows in 1e). HCN1 immunoreactivity in the knockouts was most pronounced in the middle molecular layer (MML), the termination zone of the mPP (Fig. 1k), where HCN1 signal intensity was 1.6-fold higher than the signal in wildtype animals (n = 4; p<0.001; compare Fig. 1j vs. 1k; Fig. 1n: red vs. black line). This pattern of expression resembles the HCN1 pattern seen in the perforant path axon terminals in the MML of immature, but not adult rodents [17].

**Figure 2:**
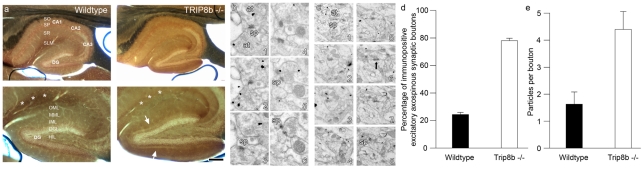
Ultrastructural localization of HCN1 in middle molecular layer (MML) of dentate gyrus. a) Light micrographs of silver-intensified immunogold-stained hippocampal slices show the presence of a thin band of HCN1 immunoreactivity in MML in the TRIP8b^−/−^ (arrows), but not in the wildtype mice. Note also the lack of HCN1 enrichment in stratum lacunosum-moleculare (SLM, asterisks) of CA1 in the TRIP8b^−/−^-section, as described by Lewis et al. [12] (see also Fig. 1d–f). b, c) Electron micrographs of serial sections showing immunopositive axonal boutons in a TRIP8b^−/−^ mouse, making both perforated (arrow) and nonperforated synapses with dendritic spines in MML. Abbreviations: at, axon terminal; sp, spine. d) Percentage of axonal boutons, immunopositive for HCN1, making axospinous synapses in MML in wildtype (black) and TRIP8b^−/−^ mice (white). e) Average number of particles per synaptic bouton in wildtype (black) and TRIP8b^−/−^ mice (white). Data were obtained from two mice of each genotype, and based on analyses of 25 synaptic boutons from each mouse (100 axonal boutons total) from the MML. Serial ultrathin sections were obtained from slices similar to those shown in (a).

To confirm that the expression of the HCN1 channels in MML of adult TRIP8b^−/−^ mice is presynaptic, we used immunogold electron microscopy to ultrastructurally localize HCN1 expression in the dentate gyrus MML. Using serial section pre-embedding silver-intensified ultrasmall immunogold electron microscopy (Fig. 2a–c), we found that ∼80% of boutons that make excitatory axospinous synapses in the MML in TRIP8b^−/−^ mice are HCN1 immunopositive, whereas only ∼24% are immunopositive in wildtype mice (Fig. 2d). Furthermore, there are only 1.6 particles per immunopositive bouton in the wildtype mice compared to 4.6 particles per immunopositive bouton in the TRIP8b^−/−^ mice (Fig. 2e). Taken together, the ultrastructural localization studies show that not only are HCN1 immunopositive excitatory axonal boutons 3-times as frequent in TRIP8b^−/−^ mice, the boutons themselves have expression levels that are nearly 3 times as high. Thus, these data replicate and extend the immunofluorescence studies and are consistent with the notion that the normal age-related redistribution of HCN1 in perforant path axons is disrupted by genetic deletion of TRIP8b.

### Normal HCN1 pattern in MML of partially TRIP8b-deficient (TRIP8b[1b-2]^−/−^) mice

Because the influence of TRIP8b on HCN channel membrane trafficking depends critically on the TRIP8b isoforms that associate with the channels [9], [10], we next wanted to know whether individual isoforms affect HCN1 expression in MML differently. For this purpose, we examined a mouse line in which exons 1b and 2 were replaced by lacZ via homologous recombination (TRIP8b[1b/^−^2]^−/−^). The removal of these exons results in absence of all except two of the TRIP8b isoforms in the brain, 1a and 1a-4 (an additional 1a-3-4 isoform can be neglected because of its low abundance in brain [9], [10]).

Immunohistochemistry using a pan-TRIP8b antibody showed that the distribution of the remaining isoforms in hippocampus corresponds to the general distribution of TRIP8b in the wildtype (compare Fig. 1a and 1g). Moreover, the intensity of TRIP8b signal was not visibly reduced in this mutant, probably reflecting the fact that together isoforms (1a) and (1a-4) represent more than 80% of all hippocampal TRIP8b protein [9]. Importantly, the hippocampal HCN1 expression pattern in mice expressing TRIP8b(1a) and (1a-4) is more similar to wildtype than TRIP8b^−/−^ mice. Specifically, HCN1 immunoreactivity is enriched in the distal dendritic field of CA1 in the TRIP8b[1b/2]^−/−^ mice (Fig. 1h), and is not significantly different from that of wildtype controls in the MML (compare Fig. 1j vs. 1l; Fig. 1n: blue vs. black line). This finding suggests that TRIP8b isoforms 1a and 1a-4 are sufficient to provide correct HCN1 trafficking in the hippocampal formation, whereas absence of one or both of these isoforms leads to mislocalization that includes disruption of age-dependent axonal trafficking in perforant path.

### TRIP8b isoform expression increases with age in entorhinal cortex

The hypothesis that TRIP8b isoforms (1a, 1a-4, or both) are critical for the age-dependent reduction of HCN1 axonal transport in perforant path predicts that 1) TRIP8b is expressed in perforant path-forming layer II stellate cells of medial EC, and 2) expression of the critical isoform(s) in EC follows a developmental gradient. To examine these questions, we performed protein and mRNA analyses of TRIP8b expression in EC. Because previous studies showing age-dependent reduction of HCN1 in perforant path have mainly been performed with rats [17], rat tissue was used for these and following experiments.

**Figure 3:**
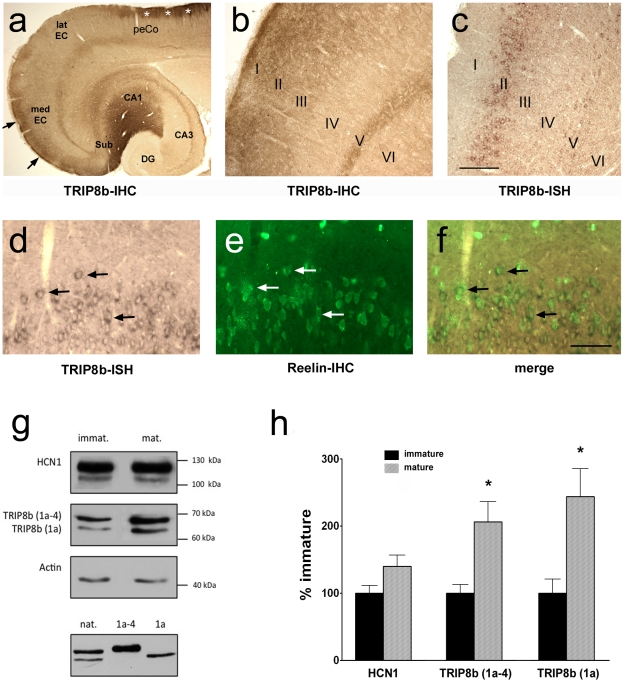
Expression analyses of TRIP8b isoforms in entorhinal cortex. a) Horizontal section showing hippocampus, entorhinal and perirhinal cortex in an adult rat, immunostained (IHC) for TRIP8b. Note: TRIP8b immunoreactivity is strong in areas which also show substantial HCN1 expression [17]: the distal dendritic fields of CA1 and subiculum (Sub) and the outer layers (I–III) of medial entorhinal cortex (medEC, arrows). TRIP8b expression is further visible in deep layer (V) of both the medial and the lateral EC, containing pyramidal cells. In the perirhinal cortex (peCo), this staining is replaced by TRIP8b immunoreactivity in the outermost layers (asterisks), most likely reflecting dendritic positioning of the TRIP8b in the pyramidal cells. b) Higher magnification view of medial EC from a): TRIP8b immunoreactivity is limited to layers I–III and V, but diffuse immunostaining does not permit identification of the TRIP8b-expressing neurons. c) Non-radioactive *in situ* hybridization (ISH) revealed expression of TRIP8b mRNA specifically in layers II and V, indicating that the stellate cells of layer II and the pyramidal cells of layer V are the major sources of TRIP8b immunoreactivity in medial EC. d–f) Co-labeling of TRIP8b mRNA (d) and reelin protein (e) further showed that many of the TRIP8b mRNA-positive cells in EC layer II co-express reelin (f), identifying them as perforant path projection neurons [25]. Scale bar: 500 µm (a), 200 µm (b, c), 80 µm (d–f). g) Upper panel: Western Blots illustrating expression of TRIP8b and HCN1 in EC tissue of immature (P10) and adult (>P60) rats. As shown previously [9], [10], probing with a pan-TRIP8b antibody revealed two bands, representing predominantly the TRIP8b(1a) and (1a-4) isoforms (∼65 and ∼70 kDa, respectively), which together constitute >80% of cortical TRIP8b. Lower panel: Native TRIP8b in EC (left) run as a control against TRIP8b(1a) (right) and TRIP8b(1a-4) (middle) harvested from HEK293 cells that were single-transfected with those isoforms. h) Quantitative analysis of HCN1 and TRIP8b isoform expression levels in immature vs. adult EC. Data are presented as “% of immature” expression and have been normalized to actin. Expression of both the TRIP8b(1a) (245±41%) and the (1a-4) isoform (200±30%) increased significantly with age (p<0.01 for both), whereas no significant change was detected for HCN1 (140±17%, p>0.05).

Detailed information about the localization of TRIP8b expression in adult EC was provided by immunohistochemistry and non-radioactive *in situ* hybridization. Immunohistochemistry with pan-TRIP8b antibody revealed a region-specific pattern of TRIP8b expression in rat EC: Immunoreactivity was dense in layers I–III (Fig. 3a, b), but this TRIP8b-signal in the upper layers was limited to the medial and vanished towards the lateral EC. Distinct TRIP8b immunoreactivity was further observed in layer V, which contains mainly pyramidal cells. This signal was seen throughout the EC and continued into adjacent perirhinal cortex, there contributing to the characteristic distal dendritic location of TRIP8b in pyramidal cells of the neocortex (asterisks in Fig. 3a; [8]). Because the cellular source of the TRIP8b expression in layers I–III of medial EC was not clearly identifiable in the immunostained sections (Fig. 3b), we additionally applied *in situ* hybridization. This technique showed that TRIP8b mRNA-expressing cells are concentrated in layer II (Fig. 3c), suggesting that the stellate cells are the main source of entorhinal TRIP8b. To further confirm that these cells are perforant path projection neurons, we combined non-radioactive *in situ* hybridization for TRIP8b mRNA with immunofluorescence for reelin, a specific marker of perforant path-forming stellate cells [25]. As shown in Fig. 3d–f, TRIP8b mRNA and reelin are frequently co-expressed in EC stellate cells, consistent with the hypothesis.

Western Blot analyses using EC tissue from immature (P10) and mature (>P60) rats were used to determine developmental time courses of the TRIP8b isoforms: These analyses regularly revealed two major bands of ∼65 and ∼70 kDa, known to mainly represent TRIP8b(1a) and TRIP8b(1a-4) isoforms, respectively (Fig. 3g; [9], [10]). Both isoforms were already detectable in immature EC, with expression levels of TRIP8b(1a) reaching ∼18% of total TRIP8b levels. Concordant with the hypothesis, TRIP8b expression in EC more than doubled with maturation (1a-4: to 200±30%, 1a: to 245±41% of immature levels; p<0.01 for both; Fig. 3h) with the proportional contribution of TRIP8b(1a) slightly increasing (to ∼21% of total TRIP8b; this difference was statistically not significant). HCN1 protein levels in EC also increased during the same time period (140±17%; Fig. 3e), confirming previous findings that the HCN1 down-regulation in perforant path with age is not associated with a down-regulation of HCN1 expression in the region of origin [17].

### Co-transfection of HCN1 and TRIP8b affects subcellular HCN1 localization in an isoform-specific manner

While the findings of the maturational expression analysis generally support a role of TRIP8b isoforms 1a and 1a-4 in the regulation of HCN1 trafficking in EC, they do not indicate what role each isoform may have. To more specifically address this question, we first used dissociated entorhinal neurons from newborn (P0) rats, which were either single-tranfected with vectors containing HCN1-EGFP, TRIP8b(1a) or TRIP8b(1a-4), or co-transfected with HCN1-EGFP and one of the isoforms. Neurons were transfected already after two days *in vitro* (d.i.v.2) and fixed two days later (d.i.v.4), in order to facilitate allocation of axons to individual neurons in an axonal network that is not yet fully developed. Transfection of these “immature cultures” resulted in overexpression of HCN1-EGFP, TRIP8b(1a-4) or TRIP8b(1a) in cells of various morphological appearances, including pyramidal- and stellate-cell-shaped neurons. About 80% of the transfected cells were neurons (as indicated by co-labeling with the neuronal marker MAP2), whereas ∼20% represented GFAP-positive glial cells (data not shown).

**Figure 4:**
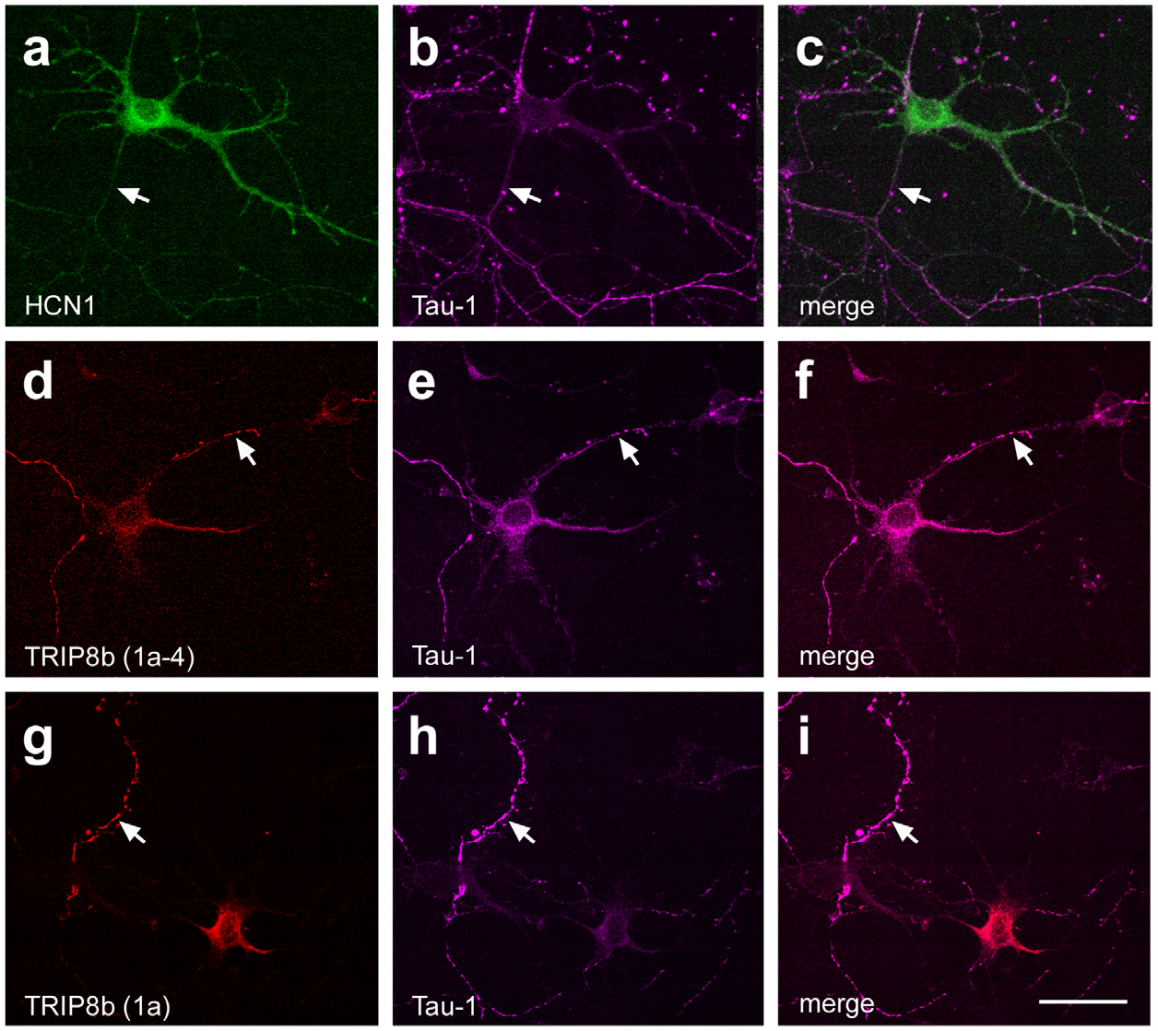
Expression of HCN1-EGFP and TRIP8b isoforms after single-transfection in immature entorhinal neuron cultures. Confocal images showing representative neurons in “immature cultures” (P0+4 days *in vitro*) single-transfected with either HCN1-EGFP (a–c), TRIP8b(1a-4) (d-f) or TRIP8b(1a) (g–i). For the identification of axons, the microtubule-associated protein Tau-1 was co-labeled (b, e, h). Note: Single-transfection resulted in a relatively homogeneous distribution of the overexpressed proteins within neuronal compartments, including the axon (arrows). Scale bar: 20 µm.

**Figure 5:**
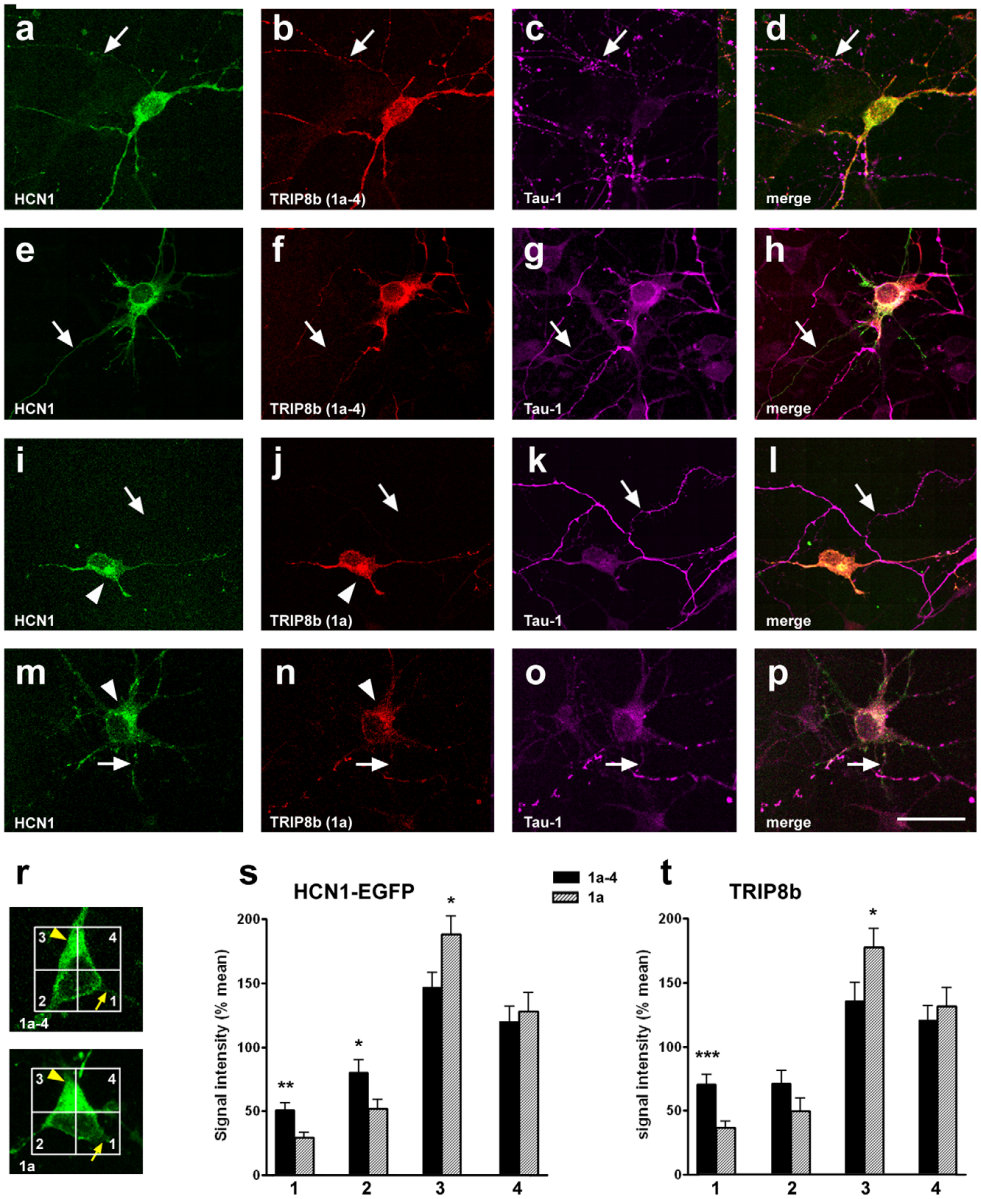
Expression of HCN1-EGFP and TRIP8b isoforms after co-transfection in immature entorhinal neuron cultures. a–h) Confocal images showing representative neurons in “immature cultures” that co-express HCN1-EGFP (a, e) and TRIP8b(1a-4) (b, f). Note: Distribution of both proteins is relatively uniform within the neurons and their expression can be detected over long range within axons (arrows) labeled with Tau-1 (c, d, g, h). i–p) Confocal images showing representative neurons in “immature cultures” that co-express HCN1-EGFP (i, m) and TRIP8b(1a) (j, n). Both proteins are not uniformly distributed in these neurons, but appear enriched in soma and proximal dendrites (arrowheads in i, j, m, n), while expression in axons (k, l, o, p; arrows) is low (m–p) or absent (i–l). Scale bar: 20 µm. r–t) Quantitative analysis of the relative subcellular distribution of HCN1-EGFP- and TRIP8b-signal in neurons co-transfected with either TRIP8b(1a) or TRIP8b(1a-4). Neuronal area was divided into four subdivisions (each 100 µm^2^) with subdivision 1 containing the axon hillock (r, arrows). Signal intensity was determined in each subdivision and relative signal intensity was calculated by dividing each subdivisional value by the mean of all four values (“% of mean”). Values from 1a-4- and 1a-transfected neurons (n = 25 each) were then compared. Note: While HCN1-EGFP and TRIP8b distribution showed a preference towards the somatodendritic subdivision 3 in both the TRIP8b(1a) and the TRIP8b(1a-4)-co-transfected neurons, enrichment in this subdivision was significantly more pronounced in neurons co-transfected with TRIP8b(1a) (HCN1: 193±14 vs. 150±12%; TRIP8b: 178±15 vs. 136±15%; p = 0.02 and 0.04, respectively). In contrast, relative signal intensities for HCN1-EGFP and TRIP8b in subdivision 1, containing the axon hillock, were significantly lower in TRIP8b(1a)- compared to TRIP8b(1a-4) co-transfected neurons (HCN1: 29±4 vs. 51±5%; TRIP8b: 36±5 vs. 70±8%; p = 0.002 and p = 0.0009, respectively).

Generally, when single-transfected, the distribution of HCN1-EGFP, TRIP8b(1a-4) and TRIP8b(1a) within the neurons was quite uniform, reaching all neuronal compartments including the axon (Fig. 4). Co-transfection of HCN1-EGFP and TRIP8b(1a-4) did not seem to markedly alter this subcellular distribution: Expression of both proteins was observed in soma, dendrites and - often over long range - in the axons of co-transfected neurons (Fig. 5a–h). The subcellular distribution was relatively homogeneous, although a slight preference for the somatodendritic compartment was observed (Fig. 5r–t). In contrast, in neurons that were co-transfected with HCN1-EGFP and TRIP8b(1a), the subcellular distribution was strikingly shifted (Fig. 5i–p), resulting in a significant enrichment of both the HCN1-EGFP and TRIP8b signal in the somatodendritic compartment (subdivision 3 in Fig. 5r), when compared to neurons that were co-transfected with TRIP8b(1a-4) (HCN1: 193±14% of mean signal intensity in TRIP8b(1a)- compared to 150±12% in TRIP8b(1a-4)-tranfected neurons, p = 0.02; TRIP8b: 178±15% compared to 136±15%, p = 0.04; Fig. 5r–t). The somatodendritic enrichment was correlated with a relative reduction of signal intensity in the area surrounding the axon hillock (subdivision 1 in Fig. 5r; HCN1: 29±4% of mean signal intensity in TRIP8b(1a)- compared to 51±5% in TRIP8b(1a-4)-tranfected neurons, p = 0.002; TRIP8b: 36±5% compared to 70±8%, p = 0.0009; Fig. 5r–t). Although this shift did not fully abolish axonal expression (Fig. 5m–p), localization of HCN1-EGFP and TRIP8b(1a) in axons appeared markedly reduced (Fig. 5i–l).

### TRIP8b isoforms may also influence the subcellular localization of endogenous HCN1 in EC neurons

**Figure 6:**
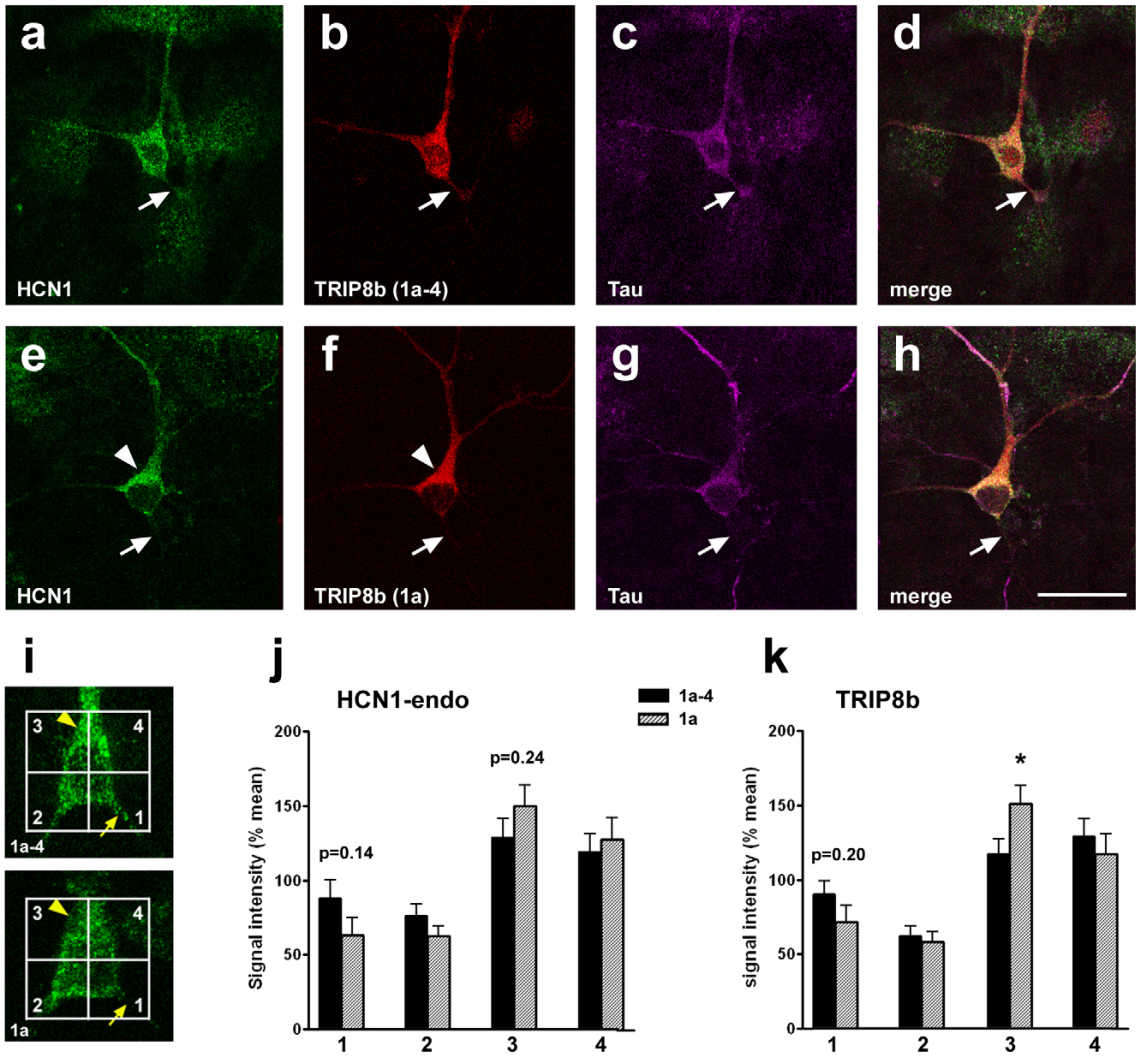
Expression of endogenous HCN1 and TRIP8b after single-transfection of TRIP8b isoforms in differentiated entorhinal neuron cultures. a–d) Confocal images showing a representative neuron in “differentiated cultures” (P5+10 days *in vitro*) that co-expresses endogenous HCN1 (a) and TRIP8b(1a-4) (b). Expression of both proteins is relatively homogenous within the neuron, involving soma, dendrites and the axon (labeled with Tau, arrows; c, d). e–h) Confocal images showing a representative neuron in “differentiated cultures” that co-expresses emdogenous HCN1 (e) and TRIP8b(1a) (f). While expression of both proteins can be observed in all compartments, including the axon (g, h), expression seems to be most intense in the somatodendritic compartment (arrowheads). Scale bar: 20 µm. i–k) Quantitative analyses of the relative subcellular distribution of endogenous HCN1- and TRIP8b-signal in differentiated entorhinal neurons transfected with either TRIP8b(1a) or TRIP8b(1a-4) (n = 25 each). Data from TRIP8b(1a-4)–overexpressing neurons confirm a relatively homogenous distribution of both the HCN1 and TRIP8b throughout the neurons (j, k). In contrast, in neurons transfected with TRIP8b(1a), TRIP8b was preferentially expressed in somatodendritic subdivision 3 (153±12 in 1a- vs. 119±10% in 1a-4-expressing neurons; p = 0.04; k). HCN1 expression in these neurons showed a trend towards enrichment in the somatodendritic compartment, which was, however, not significant (subdivision 1: 63±12 in 1a- vs. 88±12% in 1a-4-expressing neurons; p = 0.14; subdivision 3: 150±14 in 1a- vs. 129±13% in 1a-4-expressing neurons; p = 0.24; j).

Perinatally, perforant path projection neurons express only low levels of HCN1, but expression increases substantially during the second and third postnatal weeks [17]. To examine whether TRIP8b isoforms may exert similar effects also on endogenous HCN1 channels, we therefore used EC neuron cultures in a more differentiated stage. These “differentiated cultures” were prepared on P5, single-transfected with TRIP8b (1a) or (1a-4) after seven days *in vitro*, and fixed 3 days later. In these cultures, many neurons expressed detectable levels of endogenous HCN1, which localized to the soma, dendrites and axons (Fig. 6a, e, i). Overexpressed TRIP8b(1a-4) was quite uniformly distributed in these neurons and did not appear to markedly influence subcellular HCN1 distribution (Fig. 6a–d, i–k). In contrast, overexpressed TRIP8b(1a) was found preferentially in the somatodendritic compartment (153±12% of mean signal intensity, compared to 119±10 in TRIP8b[1a-4]- transfected neurons, p = 0.03; Fig. 6e–h). While the somatodendritic enrichment of TRIP8b(1a) did not result in a significant shift of HCN1 localization, a clear trend towards the somatodendritic compartment was recognizable (Fig. 6e–h, i, j, arrowheads). This suggests that TRIP8b1a may also influence the subcellular localization of endogenous HCN1 channels, although not as effective as the overexpressed HCN1-EGFP in the “immature cultures”.

## Discussion

In the present study we examined potential effects of the auxiliary subunit TRIP8b on HCN1 axonal transport using entorhinal cortex neurons (forming the perforant path) as a model. Our findings indicate that age-dependent exclusion of HCN1 from perforant path axons is dependent upon the expression of specific TRIP8b isoforms. This conclusion is based on the following observations: (1) In mice completely lacking TRIP8b (TRIP8b^−/−^), expression of HCN1 was significantly enhanced in perforant path axon terminals in the MML, compared to wildtype and to TRIP8b[1b/2]^−/−^ mice, in which expression of the major TRIP8b isoforms 1a-4 and 1a was preserved. The latter finding suggests that one or both of these isoforms prevents HCN1 from being transported to axons. (2) The developmental time course of TRIP8b(1a) and (1a-4) expression in EC, showing a strong increase with age, is in agreement with an age-dependent inhibiting effect of these isoforms on axonal HCN1 transport in perforant path [17]. (3) Finally, transfection of entorhinal neurons with TRIP8b(1a) resulted in a substantial shift of co-transfected HCN1-EGFP towards the somatodendritic compartment, suggesting that TRIP8b(1a) - rather than TRIP8b(1a-4) -, may be critical for the age-related redistribution of HCN channels in the perforant path axons.

### Role of TRIP8b for HCN1 trafficking

One of the peculiarities of HCN channels in brain is their ability to localize to different neuronal compartments depending on the type of neuron that expresses the channels. Thus, in CA1 pyramidal cells HCN1 and HCN2 channels are strongly enriched in the distal dendrites [26], whereas interneuronal basket cells interspersed between the pyramidal cells localize the same channels to somatic and presynaptic compartments [27], [28]. The subcellular trafficking of the HCN channels may be in part controlled by HCN channel binding proteins, several of which have been described [29]. However, the functional roles of most of these proteins still await clarification.

The function of one specific HCN binding protein, TRIP8b, has recently been characterized in more detail. We and others have shown that TRIP8b interacts with pore-forming HCN channel subunits to regulate channel surface expression and gating [9], [10], [11], [15], [16]. In CA1 pyramidal cells, deletion of TRIP8b significantly reduces functional I_h_ and results in loss of HCN channels from the plasma membrane, eliminating the distal dendritic gradient of channel expression [12]. However, although I_h_ was dramatically reduced, it was not completely absent in dendrites, suggesting that enrichment of HCN channels in the distal dendritic compartment, but not presence in dendrites in general, requires TRIP8b [12]. Upon further analysis of the TRIP8b^−/−^ mice, we find that HCN expression is not equally affected in hippocampal subregions. Specifically, in mice lacking all TRIP8b, HCN1 expression was enhanced rather than decreased in the dentate gyrus MML, where medial perforant path fibers terminate. This indicates that TRIP8b has region- and cell type-specific effects on trafficking and function of the HCN channels.

The molecular basis for these differential effects may lie in the extensive alternative splicing of TRIP8b at its N-terminal sequence, which contains at least two alternate translation start sites (exons 1a and 1b) followed by a variable combination of exons 2, 3 and 4. The majority of the protein, encoded by exons 5–16, is constant in all brain TRIP8b isoforms. The various isoforms exert dramatically different effects on HCN channels, some of them up-regulating and some of them down-regulating HCN surface expression, when expressed in heterologous systems or in dissociated neurons. The most abundant isoform in hippocampus, TRIP8b(1a-4), dramatically enhances surface expression of HCN1 channels, whereas other isoforms (such as the low abundant 1b-2 isoform) may have opposite effects and abolish HCN1 membrane trafficking [9], [10]. TRIP8b(1a) is the second-most abundant isoform in hippocampus and brain [9], [10], but its effect on HCN channel surface expression in neurons is not yet resolved, as Santoro and colleagues [10], [16] found a down-regulation of HCN1 current in oocytes, whereas Lewis et al. [9] observed an up-regulation of HCN1 current and surface expression in HEK293 cells. Our data from dissociated entorhinal neurons suggest that interpreting the function of TRIP8b(1a) is indeed not trivial, because normal subcellular distribution of the HCN1 channels was clearly impaired. Thus, differential expression of TRIP8b isoforms in neurons can have a significant impact on HCN channel trafficking and this impact likely depends on the cellular context.

In our study, the presence of only TRIP8b isoforms 1a-4 and 1a in TRIP8b[1b/2]^−/−^ mice was sufficient for expression of the normal pattern of HCN1, not only in DG, but generally in hippocampus, suggesting that these two isoforms are indeed the dominant TRIP8b isoforms in hippocampal formation. Because HCN1 expression in MML resembles wildtype patterns in adult TRIP8b[1b/2]^−/−^ mice, one or both of these isoforms likely plays a role in keeping HCN1 channels out of perforant path axons. According to our developmental expression analysis, both isoforms are equally likely candidates, because both show a drastic increase of expression with age. However, in our neuron culture approach overexpression of TRIP8b(1a), but not of TRIP8b(1a-4), caused a pronounced shift of the channels towards the somatodendritic compartment, suggesting that TRIP8b(1a) could be the more critical isoform that may reduce the amount of channels in axons by preferentially directing them to dendrites. Whether this involves augmented forward trafficking of HCN1 channels to dendrites or enhanced endocytosis from axonal membrane is yet unresolved. However, a role for TRIP8b(1a) in regulating the axonal trafficking of HCN1 is also supported by recent findings of Piskorowski et al. [14] showing that an artificial axonal expression of HCN1 in hippocampal CA1 pyramidal cells (which normally do not express HCN1 in axons) can be suppressed by co-expressing TRIP8b(1a), but not TRIP8b(1a-4).

Surprisingly, the effect of TRIP8b(1a) on endogenous HCN1 in the differentiated neuron cultures was not as pronounced as the effect on the overexpressed HCN1-EGFP, resulting in a trend towards, but not a significant enrichment of the endogenous HCN1 in the somatodendritic compartment. We believe that the different expression levels of HCN1 in the two experimental settings could provide an explanation for this discrepancy. Besides binding TRIP8b, which may specifically function to regulate the balance of surface versus intracellular trafficking of HCN channels [9], [10], [12], [16], proper HCN1 trafficking also depends on several other components of the cell sorting machinery [9], [16], [29], [30]. Therefore, of the endogenous HCN1 that was detected in the differentiated cultures, a substantial proportion is likely bound to other proteins and thus unavailable to interact with TRIP8b. Because these endogenous binding partners are in limited supply, such a competition should not play a major role in the neurons that overexpress HCN1, leaving a greater proportion of the channels available for specific interaction with TRIP8b.

Other aspects of our study also suggest that additional factors need to be engaged for proper channel trafficking. Thus, without HCN1 present, the capacity of TRIP8b(1a) to enter the axon is not impaired and subcellular distribution is relatively homogeneous (Fig. 4g–i), suggesting that it is the binding of the channels that alters the affection of TRIP8b(1a) for the somatodendritic compartment. This is consistent with the concept that interaction with HCNs induces conformational changes in TRIP8b [16] or alters phosphorylation states [31], [32] that allow for assembly with specific trafficking complexes. Expression of the required trafficking complexes may depend on the cellular context or on the differentiation stage of the neuron. Such a context- or stage-dependency of HCN1 trafficking could also explain our observation that TRIP8b(1a) overexpression did not always cause somatodendritic clustering of HCN1-EGFP, but allowed for a homogenous distribution of the channels in some neurons (∼10%).

In contrast to TRIP8b(1a), binding of TRIP8b(1a-4) to HCN1 did not seem to markedly affect subcellular distribution of the channels, suggesting a more general role of TRIP8b(1a-4) in promoting surface expression and membrane insertion. This interpretation partially disagrees with that of Piskorowski et al. [14], who suggested inverse roles of TRIP8b(1a) and (1a-4), with the former being mainly responsible for inhibiting axonal and the latter specifically for promoting distal dendritic expression of HCN1 in CA1 pyramidal cells. Their interpretation is mainly based on immunohistochemistry data suggesting a preferential localization of the TRIP8b(1a) isoform in pyramidal cell axons [14]. Such a location preference was not observed in our studies overexpressing TRIP8b in dissociated entorhinal neurons. When overexpressed alone, TRIP8b(1a) was found in all neuronal compartments, and in the presence of HCN1, it co-localized with the channels in soma and dendrites but rarely in axons. The use of different cell types and experimental paradigms may explain these differences.

### Functional considerations of TRIP8b regulation of HCN1 channel trafficking

HCN channels are highly sensitive to changes of the neuronal milieu, rendering them suitable to be major players in processes of neuronal plasticity [33], [34], [35], but also vulnerable to pathological dysregulation [13], [36]. TRIP8b regulation of HCN channel trafficking could contribute to both the HCN-mediated regulation as well as to the dysregulation of neuronal function. Thus, in perforant path axons, age-dependent down-regulation of HCN1 [17] and a corresponding up-regulation of Kv1.2-type potassium channels (R.A.B., unpublished observations) are likely to developmentally fine-tune synaptic properties at perforant path-granule cell synapses. Interestingly, Kv1.2 channels have been noted to be the only other channel type besides HCNs that interacts with TRIP8b [10]. It is therefore intriguing to assume that developmental changes in TRIP8b isoform expression are part of a developmental program in EC, and it will be challenging to identify other regulating factors. Neuronal activity is a likely candidate, since it has been shown to influence subcellular HCN1 trafficking in various *in vitro* systems [17], [18], [37].

TRIP8b has further been suggested to have a part in the pathological changes that occur in epilepsy. Thus, altered expression of HCN channels or I_h_ is a phenomenon regularly observed in models of experimental epilepsy [19], [20], [38], [39], [40], [41], [42], [43], and even in human epilepsy [44], and altered interaction of HCN1 with TRIP8b could be a critical mechanism contributing to this phenomen [20]. It should further be noted that mice lacking TRIP8b demonstrate motor learning deficits and enhanced resistance in tests of behavioral despair [12], indicating that a coordinated interaction of TRIP8b and HCN channels is also critical in brain regions other than the hippocampal formation.

In conclusion, our studies suggest a role of TRIP8b isoforms in regulating trafficking decisions of HCN channels in perforant path, a major afferent pathway to the hippocampus. Differential expression of these isoforms could thus constitute a new form of plasticity which could be important for processes of learning and memory [34], [45]. Notably, expression changes of TRIP8b isoforms may not only acutely affect hippocampal network activity but could also influence developmental programs, thus potentially affecting connectivity long-term [13]. Further knowledge of the regulation of these isoforms may therefore help to enhance our understanding of both physiological and pathological processes.

## Supporting Information

Figure S1 **Strategy of TRIP8b[1b/2] knock out by NIH KOMP/Regeneron.** The Pex5l gene encoding TRIP8b consists of 16 exons. An expression-selection cassette containing a lacZ reporter followed by a neomycin resistance gene driven by the human ubiquitin C gene promoter and flanked by loxP sites was inserted by homologous recombination in place of exons 1b and 2 after the 1b ATG start codon to generate the allele Pex5l^tm1(KOMP)Vlcg^.
(TIF)
