# Supplementary figures and images for: Regulation of Axonal HCN1 Trafficking in Perforant Path Involves Expression of Specific TRIP8b Isoforms
Source: PLoS One. 2012 Feb 21;7(2):e32181. doi: 10.1371/journal.pone.0032181 (PMC3283722; doi:10.1371/journal.pone.0032181)

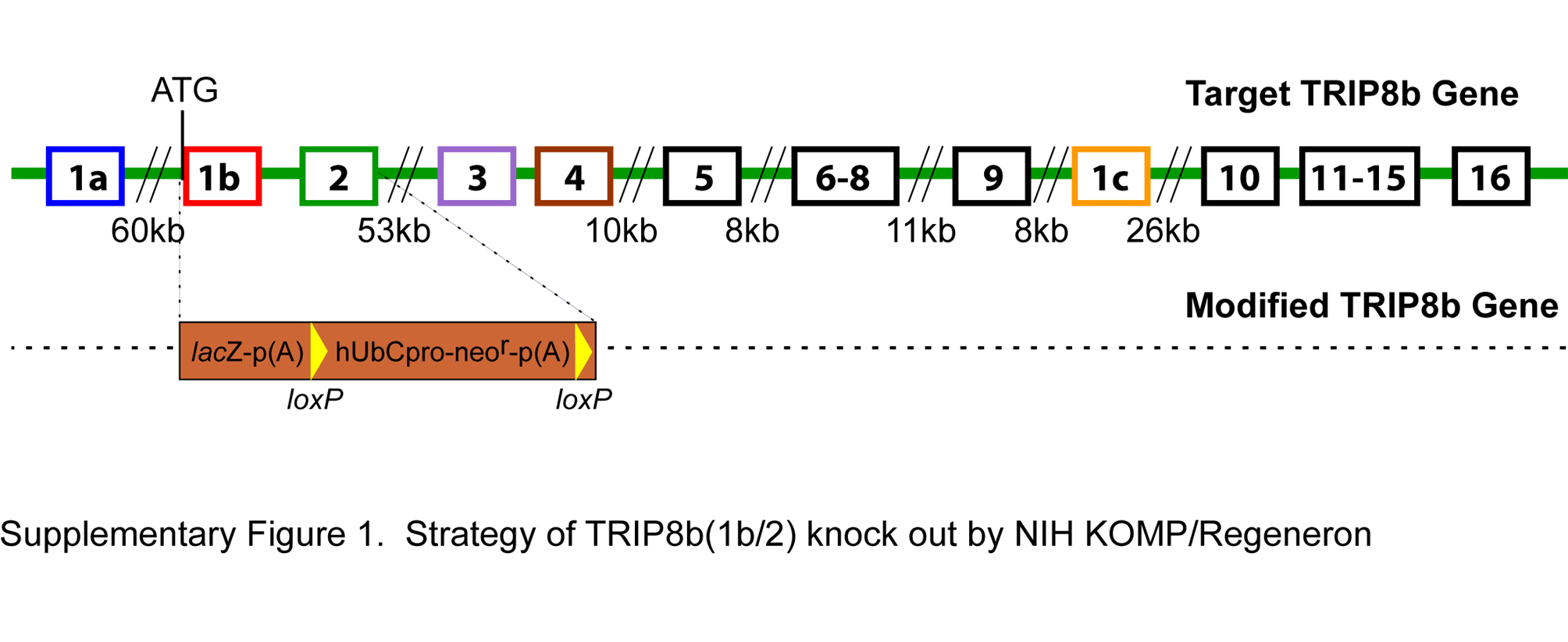

Supplement: Figure S1 — Strategy of TRIP8b[1b/2] knock out by NIH KOMP/Regeneron. The Pex5l gene encoding TRIP8b consists of 16 exons. An expression-selection cassette containing a lacZ reporter followed by a neomycin resistance gene driven by the human ubiquitin C gene promoter and flanked by loxP sites was inserted by homologous recombination in place of exons 1b and 2 after the 1b ATG start codon to generate the allele Pex5ltm1(KOMP)Vlcg. (TIF) [file pone.0032181.s001.tif]
